# Society Meetings

**Published:** 1900-09

**Authors:** 


### SOCIETY MEETINGS.

### THE BUFFALO ACADEMY OF MEDICINE.

OFFICERS—SESSION, 1900–1901.

President, Marcel Hartwig; secretary, Thomas F. Dwyer; treasurer, Charles S. Jewett; trustees, Joseph W. Grosvenor, three years; Stephen Y. Howell, two years; Benjamin G. Long, one year. The vice-presidents by virtue of being chairmen of the various sections are: Eli H. Long, chairman section of medicine; Marshall Clinton, chairman section of surgery; Albert J. Colton, chairman section of obstetrics and gynecology; Eugene A. Smith, chairman section of pathology; ; Frank W. Hinkel, chairman section of ophthalmology, otology and laryngology. The council is, therefore, composed of the aforementioned officers, the president of the Academy of the previous year, Matthew D. Mann, and the secretaries of the sections, who are: Julius Ullman, secretary section of medicine; Frederick H. Millener, secretary section of surgery; William G. Ring, secretary section of obstetrics and gynecology; Francis W. McGuire, secretary section of pathology; George F. Cott, secretary section of ophthalmology, otology and laryngology.

PROGRAM, 1900–1901.

September 4th, Surgery—Angioma, Vertner Kenerson. Resection of joints, with cases, Herman Mynter.

September 11th, Medicine—Quarterly meeting of the Academy. Clinical quantitative estimation of proteid digestion in the stomach, with demonstrations, A. L. Benedict.

September 17th, Ophthalmology—Eye-strain or gastric reflex? A. L. Benedict.

September 18th, Pathology—Modern theory of the action of the toxins and antitoxins, G. H. Clower. Blood examinations, H. G. Matzinger.

September 25th, Obstetrics. Subject and essayist to be announced.

October 2d, Surgery—Surgery among the American aborigines, A. L. Benedict. Subject to be announced, J. Henry Dowd.

October 9th, Medicine--On the involvement of the nervous system in malaria, with the report of a case of malaria presenting the symptoms of disseminated sclerosis, with necropsy and exhibition of slides, W. G. Spiller, Professor of nervous diseases, Philadelphia Polyclinic.

October 15th, Ophthalmology—Symptoms and treatment of chronic tonsillitis, Frederick H. Millener. The tonsil as a part of infection, Julius Ullman.

October 16th, Pathology—The significance of the Klebs-Löffler bacillus in the throats of persons physically well, William G. Bissell. Investigations on causation of malaria by mosquitoes, Irving P. Lyon.

October 23d, Obstetrics.—Subject to be announced, H. D. Ingraham.

November 6th, Surgery—The treatment of impotency, C. F. Durand. Surgical complications of typhoid fever, Vertner Kenerson.

November 13th, Medicine—Paresis and pseudo-paresis, Arthur W. Hurd. A case of asthenic bulbar paralysis, William C. Krauss.

November 19th, Ophthalmology—Discussion: The relation of tuberculosis of the larynx to pulmonary tuberculosis. Opened by DeLancey Rochester.

November 20th, Pathology—Prostatic hypertrophy, Herman Mynter. Pathology of stone in bladder, W. D. Green.

November 27th, Obstetrics—Subject to be announced, C. C. Frederick.

December 4th, Surgery. Quarterly meeting of the Academy. Subject to be announced, Stephen Y. Howell. Treatment of septic peritonitis, Eugene A. Smith.

December 11th, Medicine—Cyclic or periodic vomiting in childhood, Charles G. Stockton. The treatment of disease, locally, by hot air, Prescott LeBreton.

December 17th, Ophthalmology—Discussion: Strabismus and its treatment, a reconsideration. Opened by A. A. Hubbell.

December 18th, Pathology—The pathology of brain diseases, William C. Krauss. Morbid gastric physiology, A. L. Benedict.

December 25, Obstetrics—The obstetrical and gynecological clinics of Europe, P. W. VanPeyma. The kidneys and their relation to operations, Stephen Y. Howell.

January 1st, Surgery. Subjects to be announced, Chauncey P. Smith Marcel Hartwig.

January 8th, Medicine.—Arterio-sclerosis, DeLancey Rochester; Reminiscences of malaria, D. A. Morrison.

January 15th, Pathology—Sarcoid and sarcoma growths, J. C. Johnson, Assistant Pathologist, Cornell University. Urticaria, Grover Wende.

January 21st, Ophthalmology—Subject to be announced, Arthur G. Bennett.

January 22d, Obstetrics—Post-operative treatment after abdominal section, Lillian C. Randall.

February 5th, Surgery—Operative treatment of tubercular lymphomata of the neck, Prescott LeBreton. Subject to be announced, C. C. Frederick.

February 12th, Medicine—The value of clinical pathology, A. E. Woehnert. Pemphigus, with record of a case, J. W. Grosvenor.

February 18th, Ophthalmology—The inter-relationship of eye and ear diseases, E. E. Blaauw. The eye in nervous diseases, William C. Krauss.

February 19th, Pathology—Splenic anemia (splenic pseudo-leukemia), Irving P. Lyon. Leukemia, Charles S. Jewett.

February 26th, Obstetrics—Subjects to be announced, M. A. Crockett; L. G. Hanley.

March 5th, Surgery—Subject to be announced, George E. Brewer, New York City.

March 12th, Medicine—The racial factor in hysteria, Julius Ullman; Hysterial aphonia, George F. Cott. Unusual cases of hysteria, ——————.

March 18th, Ophthalmology—The making and fitting of spectacles and eye-glasses from the optician's standpoint, P. H. Meyer.

March 19th, Pathology—Quarterly meeting of the Academy. The pathology of so-called bone aneurisms, H. R. Gaylord. The principles of disinfection, H. D. Pease.

March 26th, Obstetrics—Subject to be announced, William Warren Potter. Ovarian hernia, Stephen Y. Howell.

April 2d, Surgery—Subject to be announced. Charles L. Scudder, Boston, Mass. Anemia, Edward J. Meyer.

April 9th, Medicine—Tenement house problem, Ernest Wende.

April 15th, Ophthalmology—A point in physiological optics, Lucien Howe.

April 16th, Pathology—The pathology of asthma, George N. Jack, Depew, N.Y. Retro-peritoneal sarcoma, Charles E. Congdon.

April 23d, Obstetrics—Face presentations, John V. Woodruff. Differential diagnosis between diseases of the appendix, gall bladder and right pelvic viscera, A. L. Benedict.

May 7th, Surgery—Subject to be announced, Frank W. Hinkel.

May 14th, Medicine—How are we educating our children? A. W. Bayliss; discussion opened by Prof. Henry P. Emerson.

May 20th, Ophthalmology.—Subject and essayist to be announced.

May 21st, Pathology—Investigations on trichinosis, H. U. Williams. The brachial plexus, Abram T. Kerr.

May 28th, Obstetrics—Excision of the bladder, Matthew D. Mann.

June 4th, Surgery—Subject and essayist to be announced.

June 11th,—Annual meeting of the Academy.

June 17th, Ophthalmology—Subject and essayist to be announced.

June 18th, Pathology—Changes in brain tissue in chronic brain disease, H. G. Matzinger. Remarks on etiology and pathology of epilepsy, with report of cases, Henry P. Frost.

June 25th, Obstetrics—Quarterly meeting of the Academy. Diseases of the newborn, Dewitt H. Sherman. Hemorrhagic diseases of the newborn, Irving M. Snow.

---

The Keuka Lake Medical and Surgical Association held its second annual meeting at Grove Springs Hotel, Lake Keuka, August 14 and 15, 1900. The attendance was large and the program elaborate, hence the meeting was enthusiastic. The territory embraced in the jurisdiction of the association includes the counties of Chemung, Yates, Schuyler and Ontario.

The physicians of the Lake Keuka region convened at Grove Springs last year, and that meeting was so successful in its inception and results that it was concluded to continue the meetings. The attendance at this year's association was greater by far than last year and a most decided success from every point of view. In view of that fact, it was deemed advisable to reorganise under a new name. The Keuka Lake Medical and Surgical Association was chosen, but we think it would be still better to name it simply the Keuka Lake Medical Association, the words "and Surgical" being superfluous and tending to make the name cumbersome.

Among the physicans in attendance from Buffalo were Drs. Grover William Wende and Floyd S. Crego.

The regular program was completed [at Wednesday afternoon's session and this was followed by a business meeting, at which were elected officers for the ensuing year as follows:

Henry Flood, of Elmira, president; W. Austin Macy, of Willard State Asylum, vice-president, and W. M. Smith, of Avoca, secretary and treasurer.

---

The American Microscopical Society held its twenty-third annual meeting at New York City, June 28, 29 and 30, 1900, and was a great success, both from a scientific and social standpoint.

The society received a most prompt and cordial invitation from the Denver Microscopical Society and from the Denver Academy of Science to hold its next annual meeting in Denver on the occasion of the meeting of the American Association for the Advancement of Science, August 24 to 31, 1901.

The officers elected for the ensuing year are: President, C. H. Eigenmann, Bloomington, Ind.; first vice-president, Charles M.

Vorce, Cleveland, O.; second vice-president, Edward Pennock, Philadelphia, Pa.; elective members of executive committee, C. A. Kofoid, Urbana, Ill.; John Aspinwall, New York, A. G. Field, Des Moines, Iowa.

---

THE American Electro-therapeutic Association will hold its tenth annual meeting on Tuesday, Wednesday and Thursday, September 25, 26, 27, 1900, at the Academy of Medicine, New York City, under the presidency of Dr. Walter H. White, of Boston. Members of the profession are cordially invited to attend.

---

THE Rochester Academy of Medicine was organised within the last year on lines similar to those that pertain to the Buffalo organisation. A provisional limited charter has been granted by the regents of the University, which confers the ordinary powers upon nine incorporators, who are its first trustees. Their names are as follows: William S. Ely, Eugene H. Howard, Simon L. Elsner, John O. Roe, Edward B. Angell, Edward W. Mulligan, Richard M. Moore, Charles A. Dewey and Henry T. Williams. The Academy is divided into four sections, each of which holds monthly meetings on Wednesday evenings, excepting in July, August and September, while the Academy holds regular quarterly meetings on the second Wednesday evenings of March, June, September and December. An initiation fee of $20.00 is laid which includes the dues for the first year, the annual dues thereafter being $10.00; nonresident Fellows pay $5.00. The officers for 1900 are:

President, William S. Ely; vice-presidents, Charles A. Dewey, John O. Roe, Simon L. Elsner, Richard M. Moore; secretary, Henry T. Williams; treasurer, Edward B. Angell; board of trustees, William S. Ely, Eugene H. Howard, Simon L. Elsner, Edward B. Angell, John O. Roe, Edward W. Mulligan, Richard M. Moore, Charles A. Dewey, Henry T. Williams; board of councilors, William S. Ely, Charles A. Dewey, John O. Roe, Simon L. Elsner, Richard M. Moore, Henry T. Williams, Edward B. Angell, Eugene H. Howard, Edward W. Mulligan; library committee, John O. Roe, J. Livingston Roseboom, Edward B. Angell. Section I.—Chairman, Charles A. Dewey; secretary, Charles E. Darrow. Section II.—Chairman, John O. Roe; secretary, E. Wood Ruggles. Section III.—Chairman—Simon L. Elsner; secretary, Lewis W. Rose. Section IV.—Chairman, Richard M. Moore; secretary Robert G. Cook.

The Medical Association of Central New York will hold its thirty-third annual meeting Tuesday, October 16, 1900, at Rochester. The officers for the current year are: President, John Gerin, of Auburn; vice-presidents, Lucien Howe, of Buffalo; and A. L. Beahan, of Canandaigua; secretary, Charles A. Vander Beek, of Rochester; and treasurer, S. L. Elsner, of Rochester.

---

The American Therapeutic Society was organised at Washington, D.C., May 1, 1900 The next meeting will be held at Washington, May 7-9, 1901. Dr. Eli H. Long, of Buffalo, is one of the vice-presidents.

